# A Mobile App Delivering a Gamified Battery of Cognitive Tests Designed for Repeated Play (OU Brainwave): App Design and Cohort Study

**DOI:** 10.2196/10519

**Published:** 2018-10-30

**Authors:** Martin Thirkettle, Jennifer Lewis, Darren Langdridge, Graham Pike

**Affiliations:** 1 Centre for Behavioural Science & Applied Psychology Sheffield Hallam University Sheffield United Kingdom; 2 School of Health and Related Research University of Sheffield Sheffield United Kingdom; 3 The Open University Milton Keynes United Kingdom

**Keywords:** cognitive psychology, gamification, mobile app, Morningness-Eveningness, mobile phone

## Abstract

**Background:**

Mobile phone and tablet apps are an increasingly common platform for collecting data. A key challenge for researchers has been participant “buy-in” and attrition for designs requiring repeated testing.

**Objective:**

The objective of this study was to develop and assess the utility of 1-2 minute versions of both classic and novel cognitive tasks using a user-focused and user-driven mobile phone and tablet app designed to encourage repeated play.

**Methods:**

A large sample of app users (N=13,979 at first data collection) participated in multiple, self-paced sessions of classic working memory (N-back), spatial cognition (mental rotation), sustained attentional focus (persistent vigilance task), and split attention (multiple object tracking) tasks, along with the implementation of a comparatively novel action-learning task. The “OU Brainwave” app was designed to measure time-of-day variation in cognitive performance and did not offer any training program or promise any cognitive enhancement. To record participants’ chronotype, a full Morningness-Eveningness questionnaire was also included, which measures whether a person's circadian rhythm produces peak alertness in the morning, in the evening, or in between. Data were collected during an 18-month period. While the app prompted re-engagement at set intervals, participants were free to complete each task as many times as they wished.

**Results:**

We found a significant relationship between morningness and age (*r*=.298, n=12,755, *P*<.001), with no effect of gender (*t*_13,539_=−1.036, *P*=.30). We report good task adherence, with ~4000 participants repeatedly playing each game >4 times each—our minimum engagement level for analysis. Repeated plays of these games allowed us to replicate commonly reported gender effects in gamified spatial cognition (*F*_1,4216_=154.861, *P*<.001, η^2^_ρ_=.035), split attention (*F*_1,4185_=11.047, *P*=.001, η^2^_ρ_=.003), and sustained attentional focus (*F*_1,4238_=15.993, *P*<.001, η^2^_ρ_=.004) tasks. We also report evidence of a small gender effect in an action-learning task (*F*_1,3988_=90.59, *P*<.001, η^2^_ρ_=.022). Finally, we found a strong negative effect of self-reported age on performance, when controlling for number of plays, in sustained attentional focus (n=1596, *F*_6,1595_=30.23, *P*<.001, η^2^=.102), working memory (n=1627, *F*_6,1626_=19.78, *P*<.001, η^2^=.068), spatial cognition (n=1640, *F*_6,1639_=23.74, *P*<.001, η^2^=.080), and split attention tasks (n=1616, *F*_6,1615_=2.48, *P*=.02, η^2^=.009).

**Conclusions:**

Using extremely short testing periods and permitting participants to decide their level of engagement—both in terms of which gamified task they played and how many sessions they completed—we were able to collect a substantial and valid dataset. We suggest that the success of OU Brainwave should inform future research oriented apps—particularly in issues of balancing participant engagement with data fidelity.

## Introduction

Recent advances in the performance and accessibility of Web technologies have resulted in increasing use of Web platforms to conduct cognitive psychology research. Large, diverse cohorts easily available to researchers are now accompanied by platforms capable of implementing complex tasks and accurately measuring performance [1,2]. Moving on from Web-based data collection, possibilities offered by custom built, natively coded, mobile apps include high levels of stimulus control and enormous flexibility in experimental design and data collection—both frequency of data-collecting sessions and range of data collected [3]. By collecting large sets of cognitive performance data, insights into subtle variations in cognition, both within an individual, as here, or across individuals and cultures [4], are potentially available to researchers. The aspects of tasks included here are prevalent in many everyday skills and activities—from paying attention to all potential threats when crossing a busy road (Track—multiple object tracking), to packing a suitcase efficiently (Spin—mental rotation). Understanding cognitive performance is hugely important. Even if we consider only healthy mental function, only by understanding our cognition’s fundamental properties can we design our lives [5], work [6,7], and play [8] to enable our own best performance [9,10].

A key issue for all psychology researchers is recruiting participants. While laboratory-based studies can often rely on departmental participation requirements to ensure a steady flow of—debatably—willing participants, the sample obtained is inevitably limited in demographic factors [11]. Web-based and app-based studies are one possible way of researching with a broader sample of participants, but to achieve this, researchers must ensure their task, or request, is an engaging one, especially if it requires repeated testing sessions for data collection. Embedding the experimental collecting task within an engaging, fun-to-play game is an increasingly popular way of trying to improve participant engagement and retention. A recent systematic review of gamification of cognitive tasks suggested increased engagement as one of the main reasons for gamification [12]. Moreover, this review highlighted additional benefits of gamification, such as reducing anxiety and extending the investigator’s reach, while underlining the potential that gamification has to improve data collection without necessarily impairing data’s validity.

OU Brainwave is a bespoke app, launched on multiple platforms, designed to collect research data while providing participants with understandable measures of their performances across 5 facets of cognitive ability. The app includes gamified tasks designed to measure performance on aspects of working memory, spatial cognition, sustained attentional focus, split attention, and action learning. Importantly, neither did we set out to “train” participants in any of these aspects of cognitive ability nor does the app make any promise of improvement to cognitive performance through repeated play. Instead, the app seeks to measure natural variation in performance on such tasks throughout the day [13] and in relationship to an individual’s sleep-wake cycle [14]. The app also aims to utilize a large-scale sample to answer the question of whether such variations are related to an individual’s Morningness-Eveningness score—ie, whether “Larks” perform better earlier in the day than “Owls” who perform better later [15].

Here, we present in-game data from the app, report our cohort’s broad performance across the 5 tasks in relationship to the respective task literatures and any relationships between demographic factors and performance, and discuss broader issues of gamification and task design for use in app-based testing.

## Methods

### OU Brainwave

The OU Brainwave app was designed and created in collaboration with an external developer (Conjure Ltd, London, UK). Each game included in the app went through numerous rounds of development, with usability and participant engagement given equal weight for essential factors of data validity and experimental design. The app was launched on Android and iOS mobile phone platforms in February 2015. The launch was publicized through blog posts and traditional media coverage. In addition, participants were encouraged to publicize the app through an built-in function of sharing a graph of their individual results to social media.

Ethical approval for this study was obtained from the Open University Human Research Ethics Committee. Immediately upon downloading and opening the app, participants were presented with an informed consent statement, with which they were required to agree via a tick box to continue using the app. Once consent was received, a unique participant ID for each participant was generated to link participant and future session data. Should individual participants wish to withdraw their consent at a later date, they could do so through a settings screen. Doing so deleted all participant data on the device and returned the participants to the app’s opening screen, where they had to agree again to the consent statement to reuse the app. At no point was any personal or potentially identifying information collected from the participants.

Participants entered simple demographic information: gender (male or female) and age in years although participants could choose not to answer either of these questions. Participants then completed the 5-item Morningness-Eveningness self-report questionnaire (MEQ) [16]. The MEQ is a well-established and validated research tool which measures whether a person's circadian rhythm produces peak alertness in the morning, in the evening, or in between [13,17,18], and the 5-item variation of the original questionnaire was used here to move participants to the more interactive aspects of the app as quickly as possible. Using the original scoring of this MEQ implementation, participants were coded into one of 5 types ranging from “strongly morning type” to “strongly evening type.” This result was shared onscreen with the participants, and to encourage continued and repeated participation, they were then prompted to continue to the games to “see if your performance matches your belief.”

The app also attempted to ameliorate high attrition rates from which mobile phone apps suffer by displaying the participant performance graph only once the participant had completed 3 sessions. This was made clear to participants each time they used the app until they had completed this requirement, at which point a graph of their performance, on each of the games and as an aggregate score, was shown. These graphs were designed to show participants variation in their performances on tasks across day and night, rather than to reveal absolute performance levels. As such, the performance values were normalized for each participant to highlight the best and worst scoring sessions. Accompanying the presentation of these graphs were icons encouraging the participant to share the image on social media.

At the beginning of each session (ie, on each subsequent launching of the app), participants were also asked up to 3 additional questions. A single item of mood rating [19] was included at the start of every session, “How is your mood right now?” to which they responded via a visual analog scale (VAS) slider. If a session was the first on a given day, participants were also asked what time they had woken from sleep and how many hours of sleep they had had the previous night. Participants could opt to skip answering these questions and continue to games. Each session comprised all 5 games, which were presented in a randomized order. Participants could choose to skip any game during a session, but were encouraged to complete them all through game-by-game results graphs of their individual performance within the app. These graphs were shown only after 3 full sessions to encourage a minimum level of engagement and were updated with each play after this point to promote continued play.

### Games

#### Hotspot: Action Acquisition Task

**Figure 1:**
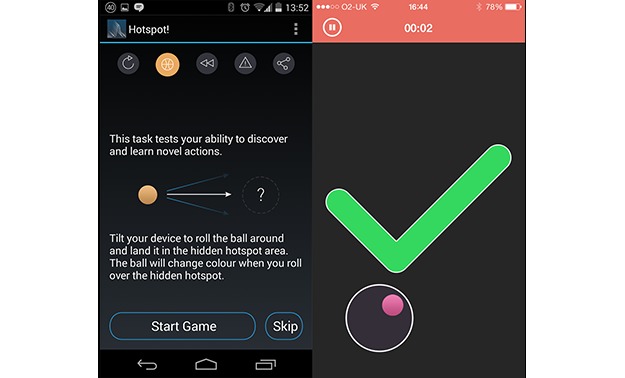
Screenshots of OU Brainwave app, showing Hotspot game instructions and play screen.

The Hotspot game is a variation on an action discovery and acquisition task [20]. In this task, participants must discover a target area by tilting their phone or tablet to roll an onscreen ball into a target area (Figure 1). The target area is unmarked, and no feedback is given until the target area is “discovered” by the participant rolling the ball over the area, at which point the ball’s color changes. Participants must then use this color change to guide them in bringing the ball to rest within the target area. Task difficulty was adjusted in development by including a 100-milliseconds delay between success (ie, entry into the target area) and a feedback signal (ie, ball’s color change). The effect of delays of this type and magnitude is to increase task difficulty [21] and was intended to prevent ceiling effects among app participants. The game consisted of 5 attempts, and each attempt presented a new, randomly chosen target area covering 5% of the game arena’s total space, with the ball covering half of that area. To succeed in the game, participants had to keep the ball within the target area for 500 milliseconds of a 1-second window. Scores were allocated so that 50% of points available were awarded for finding the target, and the remainder were apportioned according to milliseconds elapsed before the ball remained within the target area for the required time.

#### React: Persistent Vigilance Task

**Figure 2:**
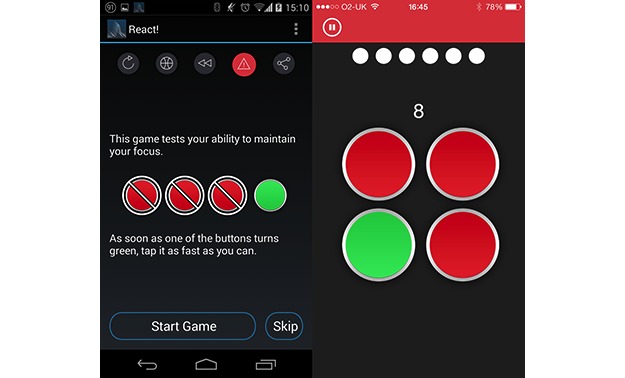
Screenshots of OU Brainwave app, showing React game instructions and play screen.

The React game was intended to be an implementation of the psychomotor vigilance task [22]. During the design process, it was decided to adjust how the task was operationalized in the game to try to increase participant engagement. This was done by including a simple choice element, which was in addition to the reaction time task and not a standard part of the classic psychomotor vigilance task. Participants were presented with 4 large, red, circular buttons (Figure 2). At a random interval from 2 to 7 seconds, one button changed color to green, and the participant had to tap the appropriate button within a 600-milliseconds window. Auditory and visual feedback was given on both correct and incorrect responses. This was repeated 8 times. Participant scores were essentially simple reaction time measures, with scores reducing according to milliseconds elapsed before a correct response was recorded, after a 100-milliseconds grace period. Responses made before the color change or incorrect button presses scored zero.

#### Spin: Mental Rotation Task

**Figure 3:**
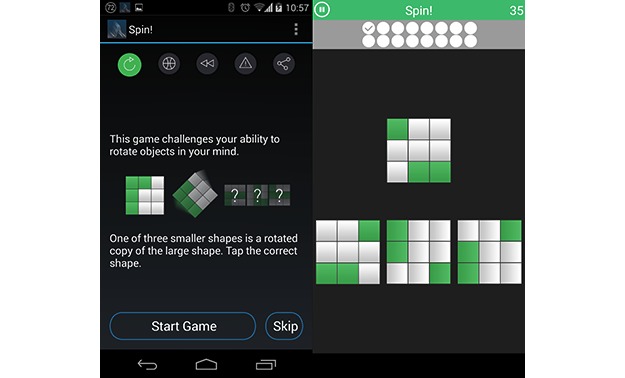
Screenshots of OU Brainwave app, showing Spin game instructions and play screen.

Spin is a gamified implementation of a spatial rotation task, using the stimulus set developed by Bethell-Fox and Shepard [23], shown in Figure 3. This stimulus set contained 18 possible patterns of filled squares within a 3×3 grid, avoiding excessive simplicity or difficulty and rotational symmetry of pattern. Each pattern contained 1, 2, or 3 groups of filled squares within the grid. While the original paper split these into levels of difficulty, all 18 patterns were presented here in a random order within a given session to provide the participant variation. In the Spin game, participants were required to match the test image with 1of the 3 options. Feedback was given in the form of ticks and crosses in circles at the top of the screen, and a timer was shown with the remaining time for the task.

Participants were presented with a large image of the target grid and had to correctly identify the rotated version of this grid from 3 alternatives presented below. The correct version was rotated at random by 90, 180, or 270 degrees. Incorrect options consisted of the test pattern reflected either vertically or horizontally. Participants had 45 seconds to make as many correct judgments as they could, up to a maximum of 18, and correct or incorrect auditory and visual feedback was given after each response.

#### Super Snap: N-Back Analog

**Figure 4:**
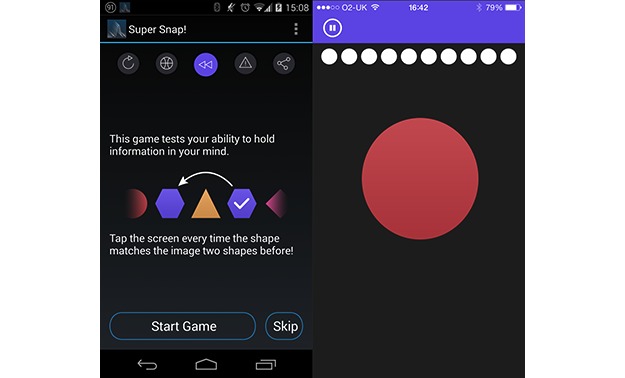
Screenshots of OU Brainwave app, showing Super Snap game instruction and play screen.

A simple implementation of the classic N-back task was used as a test of working memory (Figure 4). The N-back task has a long history of use in studies of working memory [24], see Kane [25] for a detailed discussion of the N-back’s construct validity. Here, a series of 6 brightly colored shapes (ie, circle, hexagon, rhombus, square, star, and triangle) were shown onscreen, and participants had to tap the screen to mark when the current shaped matched with previously shown two shapes. Each shape was presented onscreen for 1.5 seconds against a blank black background with an interitem delay of 1.5 seconds. Auditory and visual feedback was given after each response, along with a tick or cross at the top of the gameplay window. Participants were scored by the number of correct responses, and the game continued until 10 matches had been presented or 10 responses (including false alarm incorrect responses) had been made. Participants started each session with a score of 60 and lost 6 points for each incorrect response or miss recorded.

#### Track: Multiple Object Tracking

**Figure 5:**
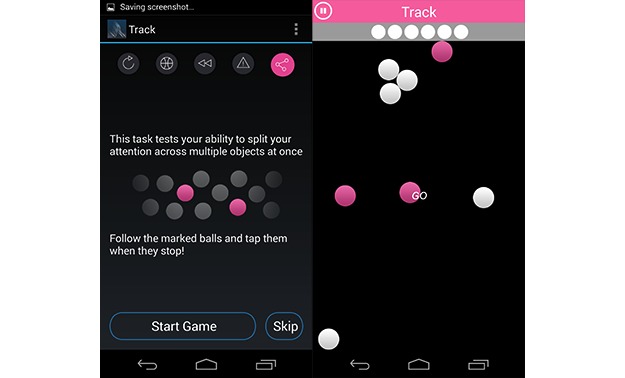
Screenshots of OU Brainwave app, showing Track game instruction and play screen.

The Track game (Figure 5) is a gamified version of a multiple-object tracking task [26]. In this task, participants had to track the location of 3 members of an array of identical moving balls. A subset of balls onscreen was highlighted before the start of the trial, before reverting to white once the trial started and all balls began to move. Participants were first shown a static array of 8, 9, or 11 balls, 3 of which were highlighted in pink, rather than the color white of the other balls. After a 3-second countdown, highlighted balls reverted to white, and all balls began moving on independent, randomly assigned trajectories. Each ball’s speed and direction of movement was adjusted randomly between each frame, and collisions between balls or borders were handled such that no ball was ever overlapped or exceeded the playing area. The balls continued in motion for 5 seconds, after which time the entire array stopped, and participants were instructed to tap the 3 balls that had been highlighted at the start of the trial. Two trials of each array size were shown, with set sizes presented in an increasing order. Participants were scored on the number of balls correctly identified with nonresponses counted as incorrect. Each correctly identified ball added a score of 2.5, so a maximum score of 45 across 6 trials was possible. Auditory and visual feedback was given after each trial, along with a tick (for correct identification of all balls) or a cross for each trial, along the top of the gameplay area.

## Results

### Demographics

#### Number of Downloads and Participants

The OU Brainwave app was launched on both Apple and Android stores on January 15, 2015. Discovery of the app peaked in its launch month, with 4394 installations during January, with the expected drop-off of installations broken only by smaller peaks in April 2015 and January 2016—both probably due to further publicity. Again, like many apps, OU Brainwave found far more users on the Apple platform than on the Android platform—with roughly two-thirds of 15,890 users across the 18.5-month data collection window using Apple devices.

Separately, and far more importantly than the raw number of downloads, is the number of active app users. As with many mobile phone apps, many downloaders either did not open the app or did not engage with the app sufficiently to be considered active users.

Of 15,890 installations between app launch and July 31, 2016, 13,979 used the app sufficiently to upload some data to the server, meaning almost 2000 downloaders did not open the app after installation. Engaging with the app for a single session only—the most popular decision among downloaders—were 3661 users, contributing at most demographic and MEQ data along with a single session’s play to the dataset. Attrition among the remaining 10,319 was predictably steep, with only 5756 users playing for >3 sessions, dropping to 1435 users at ≥10 sessions. Although no contact information was deliberately collected, so precluding any survey of participants who stopped early, potential disincentives may have included technical issues, particularly with the Android app, “pestering” by app notifications, or a perception that the app’s demands were too high.

Just over 1000 users played ≥12 sessions, and just over 100 played ≥30 sessions. Additionally, each user was free to play 1, some, or all 5 games during a given session, so while 3556 users completed 5 plays of any single game, only 2780 completed 5 plays of all 5 games. For this reason, later analyses were conducted on a game-by-game level, and no overall performance measure was calculated.

#### Demographics of Total Participant Cohort

**Figure 6:**
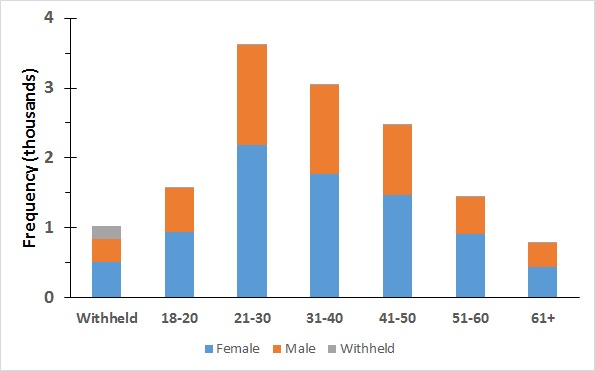
Age of participants by gender.

Of 13,979 total participants, 39.47% (5517/13,979) self-reported as male and 58.88% (8231/13,979) as female, with only 1.65% (231/13,979) declining to answer the gender question. Self-report ages of all participants are shown in Figure 6.

Distribution of participants’ reported ages shown in Figure 6 reveals a shortcoming of implementation of self-report demographic data collection. While 1033 declined to answer the age question, an exceptionally large number of participants reported their age as 18 years (965, compared with 405 for 27 years, the next most common age). While this might well be an accurate figure, it could potentially be an artifact caused by the requirement for participants to confirm they are over 18 years to use the app and play the games—a stipulation necessary for ethical approval.

Therefore, 18 years was the lowest selectable age in the self-report question. Unless age controls on downloading and installation of apps—controlled by the developer or app store rather than the end user (or parent)—become a viable option, future apps, especially particularly gamified ones, may consider collecting and subsequently discarding (or filtering not to upload) data from particular age groups, rather than attempting to exclude by self-report of age.

#### Morningness-Eveningness Questionnaire

The 5-item MEQ [16] that each participant completed produces a score from 4 to 25, ranging between extreme evening type and extreme morning type. Respondents are then traditionally classified into 5 classes by their scores (Definitely evening type: 4-7; moderately evening type: 8-11; neither type: 12-17; moderately morning type: 18-21; and definitely morning type: 22-25). From our original sample, 13,752 participants filled in all sections of the MEQ survey. As is common in studies using the MEQ, approximately half of our overall sample scored within the “neither type,” central range of the MEQ (7172/13,752, 52.2% participants). A further third of participants scored in one of the evening type categories (4584/13,752, 33.3% participants), with the remaining 14.5% (1996/13,752) scoring in the morning type categories.

**Figure 7:**
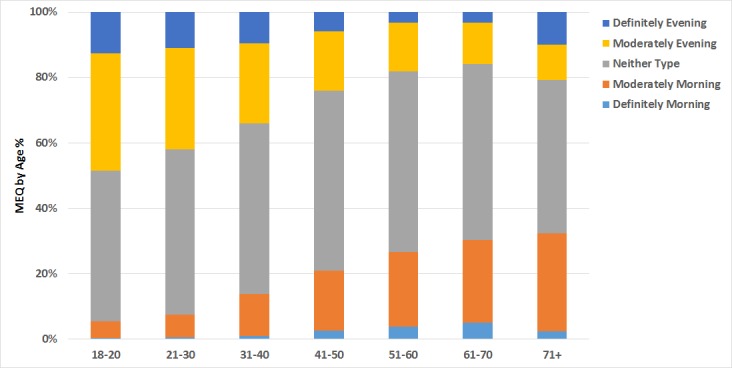
Morningness-Eveningness self-report questionnaire (MEQ) scores by age.

One of the stronger relationships usually found by the MEQ is that between age and morningness [18,27]—greater age is associated with greater morningness scores—and as Figure 7 shows, we replicated that finding here. We found a statistically significant correlation between age and MEQ score for participants who submitted both age and MEQ data (*r*=.298, n=12,755, *P*<.001). The greater absolute number of evening types than morning types in the dataset is almost certainly a result of this relationship being expressed in our cohort, which was skewed toward younger participants.

**Figure 8:**
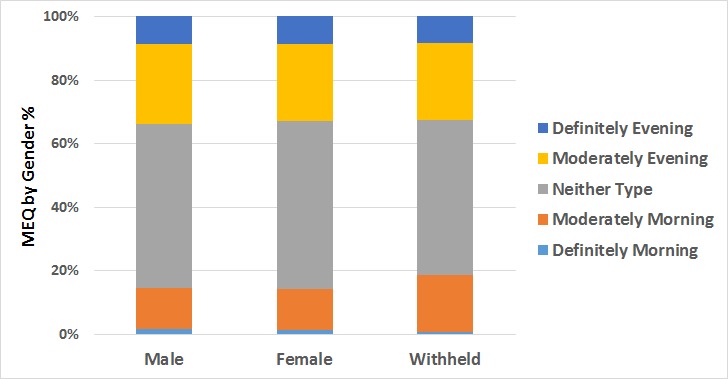
Morningness-Eveningness self-report questionnaire (MEQ) scores (color coded by MEQ category) by gender.

As Figure 8 shows, there was no significant difference between MEQ scores for males and females: mean 13.20 (SD 4.00) versus mean 13.28 (SD 3.99); *t*_13,539_=−1.036; *P*=.30. While many studies have reported greater propensity for evening types in males than in females [17], lack of gender differences reported here is not an uncommon finding in the literature [18].

### Cognitive Task Results

#### Comparison of Results to Previous Research and Demographic Effects

Because each participant could choose to play separate games individually, any 2 participants potentially played any given game a different number of times. Additionally, the full participant set includes participants who did not play a particular game sufficiently to become familiar with it. This makes handling data generated by the app very different from handling the usual data generated by tasks in OU Brainwave when conducted in a laboratory setting. To address the issue of participants who did not engage with a task sufficiently even to familiarize themselves with it, we implemented a cutoff minimum of 4 plays of each game for any participant to be included in analysis for that game. The intention was to remove participants who played no more than what would be considered a “practice trial” set in a laboratory-based experiment. However, this still leaves variability in the number of measures per participant (in terms of sessions played) and the possibility that those participants who played more would register a higher mean score on each game. Therefore, the effect on score of demographic variables was analyzed with the number of plays as a covariate. To remove outlier individuals, the most extreme 1% of average performance scores were identified and excluded before all analyses.

#### Other Effects

##### Gender

Gender effects were analyzed using analysis of covariance with the mean score of the participant as the dependent variable, gender as a fixed factor, and the number of plays as a covariate. To ensure gender and number of plays were not confounded, a *t* test was conducted to confirm no significant difference between the number of plays of a particular game by each gender. For each of the 5 games, this test was nonsignificant. Table 1 shows the mean number of plays across gender for each game and the associated *t* test statistic.

###### Track: Multiple Object Tracking

For the Track game, 4188 participants (1455 male and 2733 female) completed ≥4 sessions. Previous studies in using multiple object-tracking paradigms have shown an advantage for male participants [28], and we replicate that finding here. There was significant, but small, effect of gender on performance after controlling for number of times beyond 4 that each participant played the game (*F*_1,4185_=11.047, *P*=.001, η^2^_ρ_=.003). Male participants scored on average 1.2 points more than female participants: male: mean 38.9 (SD 12.0) versus female: mean 37.7 (SD 11.7). Although, it should be noted that variance in score accounted for by gender is very small (~0.3%) and only roughly half that accounted for by the significant effect of the number of plays beyond 4 by a particular participant (*F*_1,4174_=27.524, *P*<.001, η^2^_ρ_=.007).

###### Super Snap: N-Back Analog

For the N-back analog Super Snap, 4215 participants (1449 male and 2766 female) completed ≥4 sessions. We found no effect of gender on performance after controlling for the number of plays beyond 4 (*F*_1,4212_=2.711, *P*=.1, η^2^_ρ_=.001), although the number of plays beyond 4 was still found to improve performance significantly (*F*_1,4227_=127.06, *P<*.001, η^2^_ρ_=.03).

###### React: Persistent Vigilance Task

For React, 4241 participants (1460 male and 2781 female) completed ≥4 sessions. We found a small, but significant effect of gender on performance after controlling for the number of plays beyond 4 (*F*_1,4238_=15.993, *P*<.001, η^2^_ρ_=.004), accounting for 0.4% of variance in mean score. Male participants scored an average of 4 points more than female participants: male, mean 409.0 (SD 29.6) versus female, mean 405.1 (SD 29.9). The number of plays beyond 4 was not found to improve performance significantly (*F*_1,4238_=1.264, *P=*.26).

**Table 1:** Mean number of plays by game and gender.

Game	Number of plays, n		Number of plays, mean (SD)		*t* test	
	Male	Female	Male	Female	*t* value	*P* value
Hotspot	1403	2640	8.07 (8.56)	8.36 (7.12)	*t*_4041_=−1.150	.25
React	1495	2840	8.09 (8.52)	8.32 (7.16)	*t*_4333_=−0.933	.35
Super Snap	1459	2773	8.13 (8.52)	8.42 (7.31)	*t*_4230_=−1.175	.24
Spin	1475	2784	8.16 (8.61)	8.40 (7.36)	*t*_4257_=−0.932	.35
Track	1457	2735	8.12 (8.57)	8.38 (7.30)	*t*_4190_=−1.038	.30

###### Spin: Mental Rotation Task

For Spin, 4219 participants (1455 male and 2764 female) completed ≥4 sessions. We found significant effect of gender on performance after controlling for the effect of number of plays beyond 4 (*F*_1,4216_=154.861, *P*<.001, η^2^_ρ_=.035), accounting for 3.5% of variance in score. Male participants scored on average 3 points more than female participants: male, mean 23.6 (SD 7.7) versus female, mean 20.6 (SD 7.5). The number of plays beyond 4 was still found to have significant, albeit smaller, benefit to performance (*F*_1,4216_=60.45, *P<*.001, η^2^_ρ_=.014).

###### Hotspot: Action-Learning Task

A total of 3991 participants completed ≥4 sessions of Hotspot (1393 male and 2598 female). We found a small, but significant effect of gender on performance after controlling for the number of plays beyond 4 (*F*_1,3988_=90.59, *P*<.001, η^2^_ρ_=.022), with male participants scoring on average 3.7 points more than female participants: male, mean 31.33 (SD 12.25) versus female, mean 27.64 (SD 12.03) and accounting for 2.2% of variance in mean scores across the cohort. However, the number of plays beyond 4 was also found to have significant effect of similar size on performance (*F*_1,3988_=105.946, *P<*.001, η^2^_ρ_=.026).

In summary, of the 5 games, all but the N-back task showed significant effect of gender, after controlling for the number of plays beyond 4. In each game that showed an effect, male participants scored higher (React, Track, Spin, and Hotspot), with the strongest effect in the mental rotation-based game Spin.

##### Age Effects

For every game, there was significant positive correlation between the age of participant and the number of plays (React: n=4171, *r*=.216; Super Snap: n=4081, *r*=.221; Spin: n=4100, *r*=.216; Hotspot: n=3896, *r*=.215; Track: n=4035, *r*=.266, all significant at *P*<.001). Older participants played far more sessions than their younger counterparts, possibly due to the cohort’s self-selecting nature. Demographically, older people are less likely to either have a mobile phone or use a mobile phone for playing games [29], so it may be the case that for an older person, downloading the app represented greater commitment to engage with it. With such a confounding relationship between the number of plays and age, it was not appropriate to adopt the same approach—analysis of covariance using mean score for each participant—as to analyze gender. Instead, a stratified approach was used to analyze the effect of age on performance, controlling for the number of plays. Rather than calculating mean scores from all sessions of a participant, the mean score for each participant from only their 4th, 5th, and 6th sessions was calculated. These early snapshots provided a measure of each participant’s performance before going on to complete differing numbers of sessions and attaining an eventual average performance level.

Using this measure, we then broke participants into 7 age groups by decade (<20, 20-29, 30-39, 40-49, 50-59, 60-69, and >70). This allowed us to analyze any potential age effects with analysis of variance and to compute effect size for each game. We found significant effect of age in all games except Hotspot. Results from React (n=1596, *F*_6,1595_=30.23, *P*<.001, η^2^=.102); Super Snap (n=1627, *F*_6,1626_=19.78, *P*<.001, η^2^=.068); and Spin (n=1640, *F*_6,1639_=23.74, *P*<.001, η^2^=.080) all align with previous findings that show strong negative associations between age and reaction time [30], age and working memory as measured by the N-back task [31], and age and mental rotation [32]. The Track game, as an implementation of the multiple object-tracking paradigm, might have been expected to similarly replicate a previously found age effect for multiple object tracking [33]. While we did find significant main effect of age on multiple object-tracking performance, it was much smaller than that for React, Super Snap, and Spin (n=1616, *F*_6,1615_=2.48, *P*=.022, η^2^=.009). There was no relationship between age and performance on the action discovery task—the Hotspot game (n=1519, *F* (6,1518) = 1.78, *P*=.10, η^2^=.007). These analyses were also conducted with a correlational approach, and the pattern of results was broadly similar.

##### Mood

Before starting each session of games, participants were presented with a single mood rating to which they responded via a VAS slider. These additional data were collected to investigate the relationship between mood and cognition. Previous research has suggested a complex relationship between emotions, mood, and performance on cognitive tasks [34,35], with both beneficial and detrimental impacts on cognitive performance reported from a single, for example, positive mood induction [36]. Here, rather than inducing a given mood, we simply recorded the participant’s self-report of pre-existing mood, captured by a single item VAS measure (1-10, 10 being the happiest) [19].

To be as inclusive as possible, all participants completing ≥4 plays of each game were included in this analysis and collapsed across age and gender, giving group sizes from 3988 (Hotspot) to 4331 (React) for each of the 5 games. No strong relationship between participants’ average mood and average performance was found for any of the games, with all Pearson correlation values from 0 to.15. Similarly when correlation coefficients for every participant were calculated individually for each game, no relationship was found, with the mean rho value being <.02 in all cases, and for Super Snap, Spin, and Track, not significantly different from 0. While future analysis may explore the possibility of nonmonotonic relationships between mood and performance or potential differences in subgroups of the cohort, this first analysis suggests that either a single item VAS measure is insensitive to impactful changes of mood or that mood and performance were unrelated on any task in the app.

##### Practice Effects and Learning Curve Analysis

Each participant’s freedom to play each game uncontrolled number of times and the likelihood that this would affect an individual’s performance mean that either including number of plays as a covariate or controlling this factor in analysis is the most effective way to address a gamified testing platform’s individual freedom. However, visualizing at the group level, the effect of number of plays on performance is still possible.

**Figure 9:**
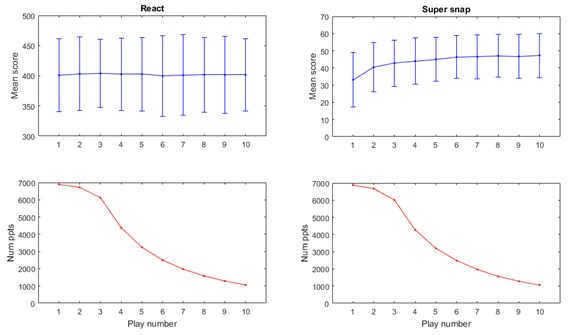
Practice effects and number of participants replaying for the "React" game and the "Super Snap" game.

Plotting mean scores for all participants in each game, except for the React game, shows typical practice effects as participants familiarized themselves with tasks. Practice effects are very common in psychological and psychometric testing and almost certainly reflect some combination of task familiarization, development of ability tested by the task, development of a strategy to complete the task as set, and possibly, reduced anxiety about the task’s mechanics [37].

While 4 of the 5 games showed typical practice effects—a stabilizing of performance following an initial rise [38]—the React game, being essentially a very basic reaction time task, seems to have been too simple to produce any practice effect-driven improvement in performance across participants’ first few sessions (see Figure 9), probably because participants immediately familiarized themselves with the task on the first play, and no effective strategies can be adopted to improve performance. However, variability of the cohort as a whole—in terms of interplay interval, age, gender, and MEQ—mean that further group analysis of practice effects is unnecessary. The presence of expected stabilization of performance after a number of plays reflects the app’s intention to measure variations in performance, rather than to train or improve participants’ abilities. Most importantly, it means that future analysis of within-individual factors (eg, time-of-day of play) should have a stable performance level from which to contrast such changes.

## Discussion

### Success of App Approaches

The large cohort collected by the OU Brainwave app, and, moreover, the repeated measurement of this cohort in quick, engaging gamified versions of classic and novel psychological tests, is another demonstration of the promise of mobile app-based testing [39]. While we deliberately did not collect more detailed demographic data, we can safely say that with such a large sample, our testing cohort would have been extremely diverse compared with samples drawn from undergraduate participant pools that typify much laboratory-based research. This, along with the sheer size of the cohort tested, should mean that any reliable findings arising from this dataset are relatively robust and not hostage to cohort effects.

The usual caveats regarding the reliability of self-report data apply to our demographic and MEQ responses [40]. While this has resulted in potential concern regarding the high number of participants self-reporting their age as 18 years, the possibility remains that this is an accurate reflection. Moreover, as a full dataset, the cohort replicates a number of age-related findings, both in increased morningness in older participants and reductions in cognitive task performance. The very low proportion of participants who withheld demographic information—only 231 participants, <2% of our total cohort, withheld either their age or gender information—is very encouraging for future mobile phone-based research, which can expect a high level of engagement from participants who download the app.

OU Brainwave is not the most downloaded research-focused app, and since its release, a number of impressively large datasets have been collected and published using other app platforms [41] and Web apps [42]. However, while OU Brainwave suffered from the same participant attrition as all apps, it recruited an impressively engaged cohort who repeatedly played games (1400 participants played >10 times) even though the games did not vary or become more challenging with continued play. This suggests that some participant engagement features were successful.

A key intention in the development of OU Brainwave was to balance the demands of behavioral experiments, in terms of data validity and operationalization of the mechanism under study, against the enjoyment and engagement of the participants. The high levels of engagement by participants who downloaded the app suggest that an in-game narrative, characters to interact with, or even an elaborate game environment may not be necessary. Studies directly manipulating the extent of gamification have reported similar lack of effect of common gamification techniques on participant attrition [43]. The games, while offering dramatically shorter sessions than one would find in laboratory testing, did not deviate far from their experimental task heritage. Except for the React game, no significant change was made from the mechanics of underlying psychological tasks, and tasks were presented without cutesy preambles, fictional scenarios, or even in-game rewards beyond simple graphing of participant performance. Withholding individual participants’ performance graphs until they had completed 3 sessions likely had the effect of carrying more participants through the steepest part of the attrition and may have contributed to the app’s longevity for these participants. Similarly, the embedded ability to share one’s performance graph—constantly updated with continued play—encouraged both the app’s spread and the individual participant’s continued engagement. Future experimental psychology apps should potentially focus on these features during app design as potentially highly effective, simple tools to encourage participation. On the other hand, our older participants’ tendency to contribute more data in terms of sessions played may suggest this group had greater intrinsic motivation to engage with the app, or at least suggest that participants’ engagement was a function of both intrinsic motivations and app features or in-game mechanics. Future studies may find it valuable to survey users during development to isolate the most valuable engagement features.

Inclusion of the full MEQ [16] and its placement at the start of the app experience meant that morningness data collected provided possibly this early analysis’s strongest finding. Our sample of over 12,500 adults revealed strong evidence for increased age correlating with morningness—with older people being more likely to be moderate or strong morning types than younger people who, in turn, are far more likely to report themselves as moderate or strong evening types. We do not replicate the finding for a similar tendency toward morningness among female respondents compared with male respondents, but as with the strong relationship found with age, this result is in line with previous studies.

Analysis of performance in the 5 cognitive tasks that make up the OU Brainwave app’s games showed that the app produced valid data and is sensitive enough to detect small but significant effects of both age and gender on cognition. We found small effects of gender in 4 of the 5 tasks (React, Track, Spin, and Hotspot) and no effect in the other task (Super Snap). In each case that a difference was found, male participants scored slightly higher on average than female participants. The greatest difference was found in the Spin game, in which gender accounted for 3.5% of variance in average score. The Spin game is a direct implementation of a mental rotation task, which has previously been found to produce large, reliable gender effects [44]. While all gender effects reported here are small, this could well be due to participants’ freedom and resulting noise in the dataset. Furthermore, the unidirectional pattern of gender effects reported here mean that an alternative explanation for these effects of platform (ie, mobile phone app), rather than cognitive task cannot be ruled out.

The impact of age on game score was much more pronounced than that of gender. Here, we reported significant reduction in game scores for older participants compared with younger participants in all but the Hotspot game and comparatively large effects in the Super Snap, Spin, and React games, in which age accounted for 6.8%, 8%, and 10.2%, respectively, of variation in average score. That we found our largest effect of age related to decline in the task most heavily reliant on reaction time is of no surprise—increases in reaction time have long been associated with increasing age [45]. However, evidence we found for adverse effect of age on both mental rotation (Spin) and working memory (Super Snap), which involve more sophisticated constructs than simple reaction time, suggests that the app is indeed sensitive to fine-grained differences in specific aspects of cognitive performance.

### Future Research

Future analysis will focus on the effect of time of day on performance. For example, those who report themselves as morning types can be compared with those who report themselves as evening types across each of the 5 tasks, although care must be taken to check and account for any bias induced by MEQ self-reporting before task performance. This approach will enable MEQ scores to be controlled or focused on in an analysis of task scores. Furthermore, recording both hours spent sleeping the previous night and time each participant awoke on each testing day will enable us to analyze the relative contribution of time spent awake, duration of preceding sleep, and time of day on any variation in cognitive performance.

### Conclusion

The OU Brainwave app, with its cohort of ~14,000 active participants represents an exciting and rich dataset. User-focused features built into the app—extremely short testing durations, allowing participants to manage their participation, engaging them through in-app feedback on their performance, and encouraging them to become an active part of the recruitment process by sharing their own performance—were largely very successful. The variability this approach introduced into the resulting performance data presented a challenge to data analysis. However, the replication of expected results and the sensitivity of the app to group-level differences in performance reported here all suggest that research apps focusing on user engagement and enjoyment, even at the expense of rigid and rigorous experimental protocols, produce valid and valuable data. Future data-collecting research apps may benefit from a similar focus on participants as users, not just as data points.

## Abbreviations

MEQ: Morningness-Eveningness self-report questionnaire
VAS: visual analog scale
